# From Vision to Reality: Five Years of the Botanical Safety Consortium

**DOI:** 10.1080/13880209.2025.2583832

**Published:** 2025-11-09

**Authors:** Constance A. Mitchell, Amy L. Roe, Scott Auerbach, Cécile Bascoul, Michelle Embry, Stephen Ferguson, Stefan Gafner, Matthias Gossmann, Bill J. Gurley, Holly Johnson, Olaf Kelber, Julie Krzykwa, Jacob Larson, Yitong Liu, Catherine Mahony, Andre Monteiro da Rocha, Stefan Pfuhler, Vincent Sica, Suramya Waidyanatha, Remco H. S. Westerink, Kristine Witt, Hellen Oketch-Rabah, Cynthia Rider

**Affiliations:** ^a^ Health and Environment Sciences Institute, Washington, District of Columbia, USA; ^b^ Procter & Gamble Healthcare, Cincinnati, Ohio, USA; ^c^ Division of Translational Toxicology, National Institute of Environmental Health Sciences, Research Triangle Park, North Carolina, USA; ^d^ Product Safety, dōTERRA International, Pleasant Grove, Utah, USA; ^e^ American Botanical Council, Austin, Texas, USA; ^f^ innoVitro GmbH, Juelich, Germany; ^g^ National Center for Natural Products Research, School of Pharmacy, University of Mississippi, University, Mississippi, USA; ^h^ American Herbal Products Association, Silver Spring, Maryland, USA; ^i^ Bayer Consumer Health, Steigerwald Arzneimittelwerk GmbH, Darmstadt, Germany; ^j^ Herbalife International of America Inc, Torrance, California, USA; ^k^ Division of Toxicology, Office of Chemistry and Toxicology, Office of Laboratory Operations and Applied Science, Human Foods Program, U.S. Food and Drug Administration, Laurel, Maryland, USA; ^l^ The Procter & Gamble Company, Mason, Ohio, USA; ^m^ Frankel Cardiovascular Center Cell Regeneration Core/Internal Medicine-Cardiology, University of Michigan, Ann Arbor, Michigan, USA; ^n^ Neurotoxicology Research Group, Division of Toxicology, Institute for Risk Assessment Sciences (IRAS), Faculty of Veterinary Medicine, Utrecht University, Utrecht, The Netherlands; ^o^ Retired, Formerly Division of Translational Toxicology, National Institute of Environmental Health Sciences, Research Triangle Park, North Carolina, USA; ^p^ Office of Dietary Supplement Programs, Office of Dietary Supplements Program (OFCSDSI), Human Foods Program (HFP) (formerly CFSAN), College Park, Maryland, USA

**Keywords:** Botanical, safety, NAMs, toxicology, natural products, dietary supplements

## Abstract

**Context:**

Botanicals, including products derived from plants, fungi, and algae, are increasingly consumed worldwide. Their complex compositions and variable phytochemical profiles present significant challenges for safety assessment. Traditional toxicology methods are time and resource intensive, and the variability of botanicals makes it difficult to test one lot as representative.

**Objective:**

The Botanical Safety Consortium (BSC), launched in 2019, was established to advance fit-for-purpose toxicity testing strategies for botanicals. This manuscript summarizes the progress of the BSC, with emphasis on the activities of its Working Groups.

**Methods:**

The BSC Working Groups evaluate established new approach methodologies (NAMs), including *in vitro* assays, in silico models, and non-protected whole organisms such as *C. elegans*, for their applicability to botanical hazard assessment. Case studies of botanicals were selected based on known toxicity profiles to test assay performance and determine whether botanicals behave differently from single chemicals in these systems.

**Results:**

The evaluations address toxicological endpoints such as hepatotoxicity, genotoxicity, developmental and reproductive toxicity, neurotoxicity, cardiotoxicity, and dermal toxicity. Early findings have identified fit-for-purpose screening tools that can generally be applied to botanical testing, with some nuances and considerations.

**Conclusion:**

Future work will focus on refining and enhancing the tool-kit through assay refinement, filling endpoint gaps with additional assays, and incorporating ADME data and in silico modeling approaches. This collaborative, science-driven framework aims to modernize botanical safety evaluation, address regulatory needs, and ultimately protect public health while supporting the global demand for botanical-based dietary supplements, cosmetics, and other products.

## Introduction

Botanicals present unique challenges for safety assessment (Mitchell et al. 2022). We define botanicals primarily as products made from plants but also including algae and fungi. Botanicals consist of dozens to hundreds of individual phytochemical constituents (Saud and Salamatullah 2021), with chemical profiles (both qualitative and quantitative) varying due to factors such as climate, growing conditions, harvesting practices, and processing methods. Even within the same plant species, geographical and environmental differences can lead to distinct chemical compositions. Compounding the natural complexity of botanicals, there is the potential for adulteration and contamination of individual ingredients, and finished products often combine multiple botanicals, making chemical characterization and safety evaluations even more challenging.

The global herbal supplement market has grown significantly this millennium, with sales increasing from approximately $4 billion in 2000 to over $12.55 billion in 2023 (Smith et al. 2024). While not common, there are known adverse effects from botanicals, such as dietary supplements or other products. For example, *Garcinia cambogia*, a tropical fruit extract promoted for weight loss, has been implicated in cases of hepatotoxicity. A widely publicized case involved the product Hydroxycut, which contained *Garcinia cambogia* as one of its ingredients. Multiple reports linked its use to acute liver injury, prompting the U.S. Food and Drug Administration to issue a warning and leading to a voluntary recall of several Hydroxycut products in 2009 (LiverTox 2012; Lunsford et al. 2016). The rising consumer demand and instances of toxicity highlights the critical need to ensure the safety of these products.

Currently safety assessments, especially in the US, presume safety for botanicals as dietary supplements (Dietary Supplement Health and Education Act of 1994 1994; Mitchell et al. 2022). Evaluations are typically based on ‘history of safe use’ (e.g., Asian ginseng having long standing evidence of safety). Evidence of harm is often needed for regulatory action and is typically identified through collecting, monitoring, and assessing adverse event reports associated with use of products that are available to consumers (i.e., pharmacovigilance) (Shaw et al. 2012; Paik et al. 2020). History of use may be sufficient for preparations that mimic traditional preparations, but often botanical supplements are a highly concentrated form of the plant or combined in a product with other botanicals. Additionally, history of use data does not usually cover delayed effects (e.g., cancer, developmental, and/or reproductive effects) and it does not account for susceptible populations (e.g., people with chronic liver disease) (Galli et al. 2019).

**Figure 1.**
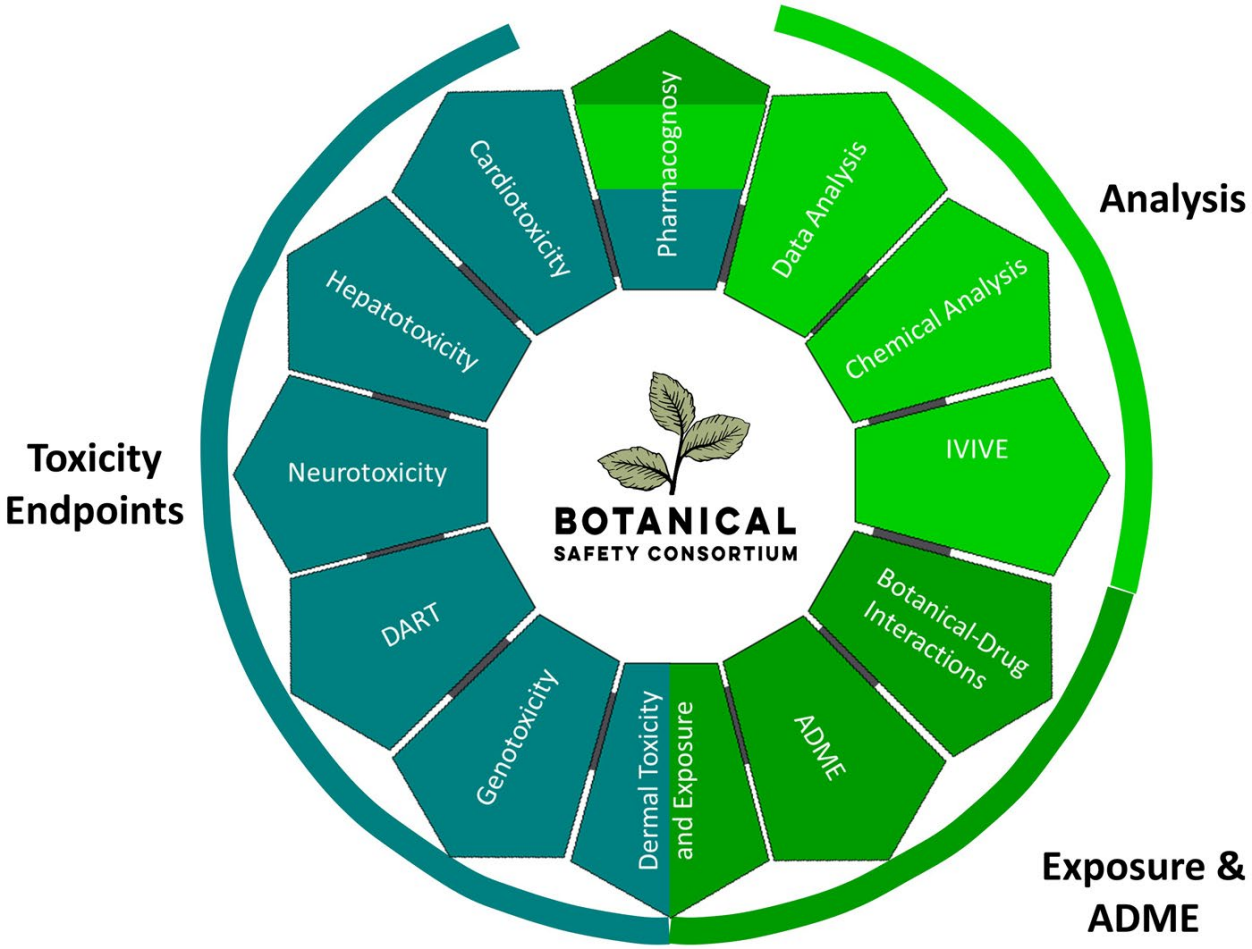
Working Groups and areas of focus of the Botanical Safety Consortium as of 2025.

There is strong consumer demand for botanical products, yet their safety assessments often fall short compared to the rigorous toxicity profiles required for pharmaceuticals and pesticides. This gap, combined with the inherent variability of botanical products, presents unique challenges. To address these, the Botanical Safety Consortium (BSC) was established as a global collaborative initiative bringing together experts from academia, industry, and government agencies (Roe et al. 2019; Mitchell et al. 2022). The BSC aims to advance the evaluation of botanical safety by identifying and evaluating fit-for-purpose toxicity assays for botanicals as complex mixtures. These tools can then be used by others to screen for toxicity. These assays encompass key toxicity endpoints, including hepatotoxicity, genotoxicity, developmental and reproductive toxicity, dermal toxicity, neurotoxicity, and cardiotoxicity (Figure 1).

The BSC is supported by experts in toxicology, pharmacognosy, chemical analysis, data interpretation, ADME (absorption, distribution, metabolism, and excretion), and *in vitro* to *in vivo* extrapolation. Its work is coordinated by a Steering Committee and executed through expert Working Groups. Officially launched in October 2019, the BSC was formalized through a Memorandum of Understanding (MOU) between the U.S. Food and Drug Administration (FDA), the National Institute of Environmental Health Sciences (NIEHS), and the Health and Environmental Sciences Institute (HESI Global) (FDA 2019).

The goal of the BSC is to identify and substantiate a suite of assays that researchers and manufacturers can use to evaluate complex botanical mixtures with a focus on human-relevant NAMs that reduce the use of *in vivo* models and are in line with the 3Rs of animal research – Reduction, Refinement, and Replacement. At this stage, the consortium is intentionally evaluating a broad suite of assays to determine which tools are most informative and reproducible for complex botanical mixtures; ultimately, the specific tools and number of assays applied will depend on their demonstrated performance, the context of use, and potential regulatory expectations. We envision that data from these assays could be useful in multiple contexts, including prioritizing botanicals for more in-depth toxicity studies, comparing related botanical products, and filling data gaps for data poor botanical ingredients. This manuscript offers an overview of the BSC’s progress since its inception in late 2019, highlighting key milestones achieved by its working groups and outlining future directions to advance botanical safety evaluation.

### Progress since 2019

Since its inception, the BSC worked to identify assays and botanicals to use as case studies. Botanicals selected as case studies (Table 1) for the assays (Table 2) were identified based on a combination of available human data (e.g., adverse event reports, clinical trials, etc.), animal data (e.g., National Toxicology Program 2-year rodent cancer bioassays) and mechanistic information (e.g., *in vitro* genetic toxicology studies). Many of the selected botanicals are not dietary supplements but make good case studies as they have known toxic effects (e.g., aconite is a known poison and is a useful case study for cardiotoxicity and neurotoxicity assays).

**Table 1.** Botanical case studies for the BSC as of June 2025.

Standardized common name	Scientific name	Plant part(s)
Aconite	*Aconitum napellus* L.	Mixed parts
Aristolochia	*Aristolochia fangchi* Y.C. Wu ex L.D. Chou & S.M. Hwang	Root
Ashwagandha	*Withania somnifera* (L.) Dunal	Root
Asian ginseng	*Panax ginseng* C.A. Mey.	Root
Blue cohosh	*Caulophyllum thalictroides* (L.) Michx.	Root and Rhizome
Comfrey	*Symphytum officinale* L.	Root or leaf
Ephedra	*Ephedra sinica* Stapf	Aerial parts
Green Tea	*Camellia sinensis* (L.) Kuntze	Leaf
Goldenseal	*Hydrastis canadensis* L.	Root and Rhizome
Kava	*Piper methysticum* G. Forst.	Root and Rhizome
Kratom	*Mitragyna speciosa* Korth.	Leaf
Milk thistle	*Silybum marianum* (L.) Gaertn.	Seed
Oleander	*Nerium oleander* L.	Aerial parts
Usnea lichen	*Usnea spp*.	Whole Plant
Tripterygium	*Tripterygium wilfordii* Hook. f.	Root
Yohimbe	*Pausinystalia johimbe* (K. Schum.) Pierre ex Beille	Bark

**Table 2.** Assays being explored and evaluated by the Working Groups.

Working Group	Assay	Status
Cardiotoxicity	Seahorse (O2 assay)	Data analysis
	Microelectrode arrays	In progress
	Voltage-sensitive dyes	Data analysis
	Transient Calcium measures	Data analysis
	Contractile Force	Data analysis
	Safety Pharmacology Screen	Data analysis
Dermal Toxicity^a^		
Developmental and Reproductive Toxicity	Transcriptomics in cell lines	Data analysis
	Zebrafish embryo mortality and development	Data analysis
	*C. elegans*	Data analysis
	devTox quickPredict assay	Complete
	Reprotracker	Planning
	Safety Pharmacology Screen	Data analysis
Genotoxicity	Ames Test	Complete
	*In vitro* micronucleus	Data analysis
	ToxTracker	Complete
Neurotoxicity	Zebrafish embryos mortality and behavioral assays	Data analysis
	*C. elegans*	Data analysis
	Microelectrode array	Data analysis
Hepatotoxicity	Transcriptomics in hepatic cells	Data analysis
	LDH release	Data analysis
	Albumin	Planning
	Cell Painting	Data analysis
	Cytotoxic reactive metabolites	Data analysis
	Cyp3A4 induction and inhibition	Data analysis

^a^ Dermal Toxicity Working Group formed in late 2023 and has not finalized their assay selection yet.

Assays (including *in vitro* assays, in silico models, and non-protected whole organism systems) were selected by each of the toxicity Working Groups based on expert opinion, availability of the assays, and endpoints covered. Preference was given to assays with some level of validation (e.g., OECD Test Guidelines). For the botanicals, a summary of expected test results is provided in Table 3. Botanicals selected as case studies were tested in the assays to assess the suitability of the assay for evaluating complex mixtures.

**Table 3.** Expected effects for each botanical in each toxicity Working Group as of 2025.

Standardized common name	Expected effects for hepatotoxicity	Expected effects for botanical-drug interaction	Expected effects for neurotoxicity	Expected effects for genotoxicity	Expected effects for cardiotoxicity	Expected effects for DART
*Aristolochia fangchi*	Unknown	Unknown	Unknown	Positive	Unknown	Unknown
Ashwagandha	Unknown	Unknown	Negative	Unknown	Unknown	Unknown
Asian ginseng	Negative	Positive	Negative	Negative	Negative	Negative
Blue cohosh	Unknown	Unknown	Unknown	Unknown	Unknown	Positive
Comfrey	Positive	Unknown	Unknown	Positive	Unknown	Unknown
Ephedra	Positive	Unknown	Positive	Unknown	Positive	Unknown
Goldenseal	Positive	Positive	Unknown	Negative	Negative	Positive
Green tea	Positive	Positive	Negative	Mixed evidence	Unknown	Unknown
Kava	Positive	Unknown	Negative	Negative	Unknown	Unknown
Kratom	Positive	Positive	Positive	Unknown	Positive	Unknown
Milk thistle	Negative	Positive	Negative	Mixed evidence	Negative	Unknown
Usnea lichen	Positive	Unknown	Negative	Mixed evidence	Unknown	Mixed evidence
Yohimbe	Mixed evidence	Unknown	Positive	Unknown	Positive	Unknown
Tripterygium	Unknown	Unknown	Positive	Unknown	Positive	Unknown
Aconite	Unknown	Unknown	Positive	Unknown	Positive	Unknown
Oleander	Unknown	Unknown	Positive	Unknown	Positive	Unknown

Note, more details including literature are published in each Working Group’s strategy paper [hepatotoxicity and botanical-drug interactions (Roe et al. 2025); neurotoxicity (Kanungo et al. 2024); genotoxicity (Witt et al. 2025); cardiotoxicity (krzkywa et al. 2025); DART (Mahoney et al. Under Review)]. green indicates positive, blue indicated negative, salmon indicates mixed evidence, gray is unknown. Additionally, some Working Groups are considering additional botanicals to better understand their assays (e.g., DART and dermal).

Additional foundational work was done to source and provide authentication evidence for the botanicals selected, to ensure that responses in assays were due to expected constituents not adulterants or mistaken identity (Waidyanatha et al. 2024). Work was also done to collect existing literature on the botanical case studies, resulting in a best practices guidance and template for conducting literature reviews on botanicals (Patel et al. 2023). Additional work is underway to incorporate ADME information to help aid in the interpretation of results from NAMs. Finally, progress on the data analysis process is described to evaluate the assays and what further work needs to occur.

Each section below describes the progress and planned next steps for each Working Group. Every team has shared their strategy and preliminary results at scientific conferences for feedback, including meetings like the Society of Toxicology, the International Conference on the Science of Botanicals, or meetings relevant to their focus (e.g., Safety Pharmacology Society Annual Meeting for Cardiotoxicity).

## Chemical Analysis

The Chemical Analysis Working Group has been crucial to the success of the BSC, providing evidence for the authenticity of botanical materials tested in the bioassays (Waidyanatha et al. 2024). Additionally, by identifying and quantifying key constituents, this enabled in silico modeling for toxicity endpoints and ADME.

Botanicals were selected by the toxicity Working Groups based on existing data (positive and negative) from toxicity studies. Most botanicals were sourced in large quantities (1–2 kg) to provide sufficient test sample of the same material in each assay. Sources were selected based on expertise in the Working Group and often came with certificates of authentication. No commercial ‘off-the-shelf’ products were used in this phase (e.g., capsules from a wellness store). For the BSC, the majority of extracts were prepared at the National Center for Natural Products Research (NCNPR), University of Mississippi (Gurley et al. 2025). Extraction methods reflected profiles reported in the literature, with 95% ethanol used to capture key constituents. Solubility testing was done in ethanol and DMSO, and most extracts were equally or more soluble in DMSO. Since DMSO is commonly used in *in vitro* assays, it was selected as a vehicle control in the assays and used to prepare the dose formulations for the assays.

Analytical methods, including liquid chromatography and mass spectrometry, were used to identify and quantify constituents. While one lot of each botanical was used in the bioassays, multiple lots were analyzed by the Chemical Analysis Working Group for green tea and ashwagandha as pilot studies to compare constituent quantities (Sica et al. 2025). For example, 3 lots of green tea extracts were screened to select one lot which was characterized using commercial standards, identifying major compounds totaling approximately 81% of extract weight (Waidyanatha et al. 2024). Other methods like high performance thin layer chromatography (HPTLC) were used as orthogonal results to support authentication of the botanicals. Additionally, contaminant analyses were performed in select cases to support that effects induced in the assays are due to the botanical itself, not a heavy metal or microbial contamination. Data are publicly accessible *via* the National Toxicology Program (NTP) website [https://cebs-ext.niehs.nih.gov/cebs/paper/15717] (NTP 2024). Additionally a manuscript is published describing the strategy of the Working Group with more details (Waidyanatha et al. 2024). There is also work to publish methods on the constituent quantification, including milk thistle (Muchiri and van Breemen 2024) and kratom (in progress) chemical standardizations and another on ashwagandha incorporating a semi-universal detector (Sica et al. 2025).

Future work for this team will include publishing additional methods papers, supporting assay data analysis, and expanding the botanical library as needed (e.g., for the Dermal Working Group or adding more botanicals to other Working Groups).

## Literature review process

Toward the mission of the BSC, current assay results will be benchmarked with existing literature on botanicals. Therefore, to ensure information was collected in a systematic way, a literature review process was created, including the development of a template that other researchers doing botanical toxicity research can use and was published in an open-access publication (Patel et al. 2023). Sections in the template include pre-clinical and clinical data, accepted nomenclature, traditional use, commercial use, and known constituents. By fostering collaboration between toxicologists, pharmaconogists, and botanists, this review process supports the collection and generation of high-quality research, which can help advance the safety evaluation of botanical products.

The literature review process was developed as a structured framework for consolidating data, resources, and best practices to inform botanical safety studies efficiently and comprehensively. Once the process was completed, toxicity Working Groups used the template to collect information on the selected botanicals to later benchmark assay results to existing literature.

## Data Analysis

The Data Analysis team plays a central role in standardizing and interpreting the large amount of data generated by the various Working Groups. The team developed a standard data template to collect information from disparate labs and formats in one form, enabling easier comparison of data across assays and botanicals.

A uniform data analysis pipeline was developed as a first pass of analysis so that working groups could get a sense of results and how the assays compare to existing literature on specific botanical ingredients. Benchmark Dose Modeling (BMD) was used to determine whether a botanical was ‘active’ in an assay across the tested concentrations. Data analysis will be an iterative process with the involvement of data analysts and Working Group members to understand the typical analysis methods and statistical tests associated with the assays. For example, any statistically significant increase in cytotoxicity may be worth noting for ‘activity’ but in order to determine specific toxicity to a given system, additional biological changes could be required.

The Data Analysis team’s primary role is to provide a standardized framework for data interpretation, rather than making definitive determinations about activity or inactivity for the botanicals. This iterative process relies on close collaboration with assay developers, ADME specialists, and other experts to align analytical methods with the biological relevance of the models. Looking ahead, the team is focused on analyzing increasingly complex datasets, such as multi-dimensional data from zebrafish embryo behavioral studies. Additionally, the team aims to publish its pipeline, compare BMD modeling with pairwise comparisons of the datasets, and make data publicly available to promote transparency.

## Hepatotoxicity

The Hepatotoxicity Working Group is developing screening strategies to identify botanicals with potential hepatotoxic effects, both directly and due to botanical-drug interactions. This team is exploring the use of in silico tools and *in vitro* assays, using botanicals as case studies to evaluate the utility of the tools. A full description of the assays and botanicals selected can be found in the 2024 publication of the Working Group (Roe et al. 2025).

Botanicals were selected as case studies for hepatotoxicity based on evidence from clinical studies and animal studies. These include comfrey, goldenseal, decaffeinated concentrated green tea, kava, and kratom, all of which have demonstrated varying degrees of liver-related injuries. For example, comfrey is linked to sinusoidal obstruction syndrome due to pyrrolizidine alkaloid metabolism and endothelial cell damage (Mei et al. 2010), while goldenseal has been associated with liver tumors and cholestatic hepatitis in animals (NTP 2010). Green tea extracts, particularly when catechins are concentrated, have been implicated in hepatotoxicity under fasting conditions in animal studies (Oketch-Rabah et al. 2020).

The group has explored various *in vitro* assays to screen for hepatotoxicity and botanical drug interactions. For cell types, primary human hepatocytes were selected for initial work, both as two-dimensional human hepatocyte cultures and hepatocyte spheroids, though other cell types (e.g., HepG2 or HepaRGs) were initially discussed. Various assays were selected including ‘traditional’ *in vitro* endpoints for liver like LDH leakage, albumin measurements, and CYP 3A4 induction and inhibition. Additionally, more ‘omics based approaches like Cell Painting and transcriptomics were utilized to provide both mechanistic information and cell-population level effects.

Preliminary results have been promising when comparing assay results to existing data. For example, hepatocyte models have demonstrated LDH leakage and decreased albumin production for botanicals like kratom, while Cell Painting data have revealed patterns indicative of cellular stress for goldenseal. Additionally, a session organized by this team at the 2024 Society of Toxicology meeting was published as a workshop report, highlighting liver induced toxicity *via in vitro* studies, clinical trials, and adverse event reports (Koturbash et al. 2024).

Future efforts have been discussed to include expanding the assay battery to include phase 2 enzyme induction and inhibition assays, oxidative stress assays, and deeper analyses of omics data. The group also is considering the evaluation of multiple lots of specific botanicals, studies of individual phytochemical constituents, and the incorporation of gut models to better understand botanical interactions. Preliminary results are also guiding potential follow-up work. For example, kava constituents showed substantial induction of CYP3A4 *in vitro*, which to our knowledge has not been previously reported. To see if this effect is true or an *in vitro* over prediction, the group is considering measuring internal doses using other models to better approximate how this extract would be processed in the human gastrointestinal systems.

## Genotoxicity

The Genotoxicity Working Group is developing a screening strategy to identify botanicals with genotoxic potential. Similar to the Hepatotoxicity Working Group, existing assays for genotoxicity were selected for testing with data-rich botanicals. A full description of the team’s strategy was published in early 2025 (Witt et al. 2025).

Botanicals selected by this group have a range of genotoxic and non-genotoxic activity. For instance, Aristolochia was selected as a prime candidate for assay evaluation because of its well-documented genotoxicity attributed to its major constituents- aristolochic acids e.g., (Bhalli et al. 2013). Comfrey was also selected as a case study expected to yield positive results in genotoxicity assays, as it contains pyrrolizidine alkaloids which are documented genotoxicants (EFSA 2011). Additional botanicals such as goldenseal root, kava root, and milk thistle provide a spectrum of genotoxicity and carcinogenicity profiles that are expected to help in refining testing approaches.

The Working Group was able to select from many well-established *in vitro* assays for evaluating genotoxicity. The initial strategy is based on using the bacterial reverse mutation (Ames) test (OECD 2020) and two *in vitro* micronucleus assays (OECD 2023), each in a different mammalian cell type, to evaluate 13 of the botanical case studies. These two types of assays cover gene mutations and chromosomal damage, respectively. Additionally, the ToxTracker^®^ assay (Hendriks et al. 2016; 2024) is being used to identify induction of DNA damage and certain types of cellular stress. In addition to the *in vitro* assays, in silico genotoxicity predictive modeling of identified constituents of the 13 botanicals has been conducted.

Initial test results are promising. Expected positives were identified by the combination of assays and expected negatives were confirmed. For example, Aristolochia induced positive responses in all four assays as expected. In contrast, comfrey, another known positive, induced a less potent response in the Ames test than expected, possibly due to the concentration of pyrrolizidine alkaloids in the extract. To clarify the results with comfrey, efforts are underway to benchmark the genotoxicity of the individual pyrrolizidine alkaloids against the results from the whole-extract. Constituent versus whole-extract comparison of genotoxicity will be extended to include all 13 botanicals. Testing with the ToxTracker assay has provided complementary data to the Ames and micronucleus assays, enhancing insights into the mechanistic pathways of genotoxicity of the 13 botanicals.

Future efforts will include follow-up testing to extend our knowledge about the performance of the testing battery and about the relevance of findings in the context of extracts with previously unknown genotoxicity potential. In select cases, to further contextualize results, advanced methods like error-corrected sequencing for mutagenicity assessments may be utilized. The group also aims to refine data interpretation by integrating in silico toxicity predictions with experimental data. Collaborations with the ADME team will explore how metabolic pathways influence genotoxicity, improving the interpretation of in silico and *in vitro* findings. Additional data analysis will include benchmark dose modeling for *in vitro* micronucleus data and leveraging the Tgx-DDI transcriptomic biomarker (Li et al., 2019) to gain further insights into DNA damage pathways.

In addition to the strategy paper (Witt et al. 2025), discussions on study design for the Ames test resulted in a publication exploring whether repeat tests are necessary, which is a benefit to the entire field of genotoxicity testing (Zeiger et al. 2024).

## Developmental and Reproductive Toxicity (DART)

The Developmental and Reproductive Toxicity (DART) Working Group is developing a strategy to screen botanicals for DART potential. By employing a combination of *in vitro*, and non-protected animal models, together with characterization data and in silico tools, the group is evaluating whether these methods can reliably assess the DART potential of botanicals. A more in-depth description is available *via* Mahoney et al. (under review).

Similar to other Working Groups, botanicals for DART assays were selected based on existing data, including animal studies and mechanistic information. Blue cohosh, traditionally used to induce labor, has been associated with fetal cardiac defects, craniofacial abnormalities, and mitochondrial impairment (Rader and Pawar 2013). Asian ginseng was chosen as a negative control due to its evidence of a lack of reproductive or developmental toxicity (CIR 2012). Other botanicals have disparate evidence on DART.

The group is exploring the combination of various tools to screen for DART, including human pluripotent stem cell biomarker studies, zebrafish embryo assays for mortality and malformations, *C. elegans* reproduction and development assays, safety pharmacology assays related to DART targets, and transcriptomics in both zebrafish embryos and human cell lines for Connectivity Map (CMap) analysis and mechanistic insights. Preliminary results indicate a higher activity for blue cohosh versus Asian ginseng in the DevTox quick predict model, with evidence also of malformations in zebrafish embryos and fecundity in *C. elegans* respectively. Connections were observed in CMap analysis of the blue cohosh transcriptomics data to substances of known teratogenic and developmental risk and in the safety pharmacology panel, there was evidence of interactions with a number of targets that associate with more potent developmental toxicants.

Next steps will include a deeper analysis of current data collected for the botanical case studies, including integration of biomarker, functional and mechanistic data and considerations of the limitations of the various assays. Concentration responses and aspecific effects of high doses has been identified as an area where more evaluation is needed. The group is also exploring characterization data and QSAR tools such as the DART Decision Tree.

It was difficult to find plants related to dietary supplements with reported or suspected DART effects, and so following these initial case studies, the group plans to expand the case studies to include additional botanicals, particularly those known to be associated with issues in livestock (e.g., *Veratrum californicum;* Welch et al. 2011). Notably, even though the goal eventually is to be able to screen across the entire reproductive lifecycle, such botanicals are generally more associated with adverse effects on embryo development and given this is of paramount importance, these will be prioritized.

Finally, exposure considerations (e.g., placental transfer, fetal exposure, breast milk exposure) and evaluation of NAMs that were not available at the start of the program have been identified for further exploration.

## Cardiotoxicity

The Cardiotoxicity Working Group is focused on evaluating *in vitro* tools to screen for the cardiotoxicity of botanicals. The group has conducted studies on 16 botanicals, including aconite, ephedra, and oleander, which were selected based on their known cardiotoxicity. Aconite, for instance, has been linked to clinical disorders such as tachycardia (Majumder et al. 2023), making it a compelling case study for cardiotoxic evaluation. A full description of the assays and botanicals selected by this team can be found in Krzykwa et al. (2025).

The group employed human induced pluripotent stem cell-derived cardiomyocytes (hiPSC-CM) for the selected assays, given their established use in cardiotoxicity testing. Assays included microelectrode arrays (MEAs) (Sala et al. 2017), optical mapping for calcium and voltage changes (da Rocha et al. 2020), and methods to measure mitochondrial function (Holt et al. 2023) and contractile force (Goßmann et al. 2020). These techniques assessed parameters such as action potential characteristics, contractile behavior, and rhythm irregularities.

Preliminary results demonstrated that yohimbe exposure resulted in prolonged calcium cycling, implicating its effects on the sarcoplasmic reticulum calcium pump. Green tea exhibited dose-dependent reductions in contractile activity and a complete loss of network synchronization at high concentrations. In contrast, ginseng showed minimal effects under the same conditions, suggesting a low cardiotoxic potential. Some notable challenges with these assays were solubility issues and determining appropriate concentration ranges for testing so that many botanical extracts produced effects only at high concentrations.

Future efforts will focus on refining concentration ranges, comparing findings across other cell types, and integrating in silico predictions with experimental data. The group also plans to collaborate with external teams working on safety pharmacology and advanced 3D cardiac models to enhance the translational relevance of their findings. Moving forward, an aim is to incorporate more demographically diverse cell lines to evaluate differences in botanical-induced cardiotoxicity across sexes and ethnicities. By exploring donor pools from diverse populations, the group hopes to better understand variability in cardiotoxic responses and improve the applicability of their findings to real-world scenarios (Paik et al. 2020).

## Neurotoxicity

The Neurotoxicity Working Group is evaluating NAMs as potential screening tools for neurotoxic endpoints in botanicals. Botanicals were identified based on their suspected neurotoxicity, with a focus on known mechanisms of action, *in vivo* studies, human clinical data, and activation of neuronal pathways. Examples include aconite and oleander, both of which are known to impact neuronal function (Farkhondeh et al. 2020; Li et al. 2022). A full description of the strategy for this Working Group has been published (Kanungo et al. 2024)

The group is exploring multiple assays and endpoints to evaluate neurotoxicity. MEAs were used to measure electrical activity in cortical neurons, capturing parameters such as spikes, burst activity, and network synchronization (van Kleef et al. 2024). Zebrafish embryo behavioral assays provided insights into sensory-motor responses, while *C. elegans* locomotion assays assessed movement and response to stimuli. These complementary methods enabled the group to evaluate the impact of botanical compounds on various aspects of neuronal function, ranging from electrical activity to organism-level behaviors.

Preliminary findings demonstrated dose-dependent decreases in neuronal activity for botanicals such as ephedra. Aconite exhibited a distinct phenotype characterized by intense but diminished bursts of neuronal activity, likely linked to its modulation of sodium channels. Ginseng, in contrast, showed minimal effects, suggesting low neurotoxicity. Despite these promising results, challenges remain in interpreting assay results and correlating *in vitro* effects with *in vivo* relevance. Discrepancies between predicted and observed activities highlight the need for further exploration of dose levels, metabolism, and the ability of compounds to cross the blood-brain barrier.

Future efforts will focus on enhancing data analysis, including the application of benchmark dose modeling across multiple endpoints to better characterize neurotoxic effects. The group is exploring the addition of acetylcholinesterase activity assays to capture critical pathways not currently addressed in their battery of tests. Assays targeting developmental neurotoxicity are also under consideration, as they may provide a more comprehensive understanding of the impact of botanicals on neuronal development and function. The group has noted the potential for alignment between cardiotoxicity and neurotoxicity assays, given the shared characteristics of excitable cells in these systems. This overlap may present opportunities to streamline testing for both endpoints, improving efficiency while maintaining scientific rigor. Additionally, zebrafish embryos and *C. elegans* were utilized by this team and by the DART Working Group, another opportunity to compare endpoints. By integrating results from complementary tools and addressing gaps in their current assay battery, the Neurotoxicity Working Group aims to enhance the evaluation of botanical neurotoxicity and contribute to the broader understanding of botanical safety.

In addition to the strategy paper, this team has published results from the MEA studies (van Kleef et al. 2024; Van Kleef et al., 2025).

## Absorption, distribution, metabolism, excretion (ADME)

To enhance the interpretation of *in vitro* toxicity study results, ADME modeling is being piloted in the BSC for the individual constituents quantified by the Chemical Analysis team. Using tools originally developed for pharmaceuticals, these methods assess the physicochemical properties, metabolism, and toxicity potential of botanicals, providing critical context for bridging *in vitro* findings to broader toxicological evaluations.

The team analyzed 103 phytochemical constituents from 13 botanicals to predict properties like solubility, permeability, absorption and clearance pathways. Commercially available tools like ADMET Predictor^®^ classify these compounds using frameworks such as the Biopharmaceutical Classification System (BCS) and Extended Clearance Classification System (ECCS). The BCS groups compounds by solubility and gut permeability, helping to interpret bioavailability data. For instance, glycosides from ashwagandha and Asian ginseng show low permeability (BCS class III or IV), leading to poor absorption and bioavailability (Liu et al. 2024). The ECCS categorizes clearance pathways, revealing that many phytochemical constituents, including those found in ashwagandha, blue cohosh, ephedra, goldenseal, green tea, kava, kratom, and yohimbe are classified under ECCS class 2, whose clearance is primarily mediated by liver metabolism and exhibit high interaction potentials with cytochromes P450 enzymes (CYPs). (Liu et al. 2025). This work further highlights the interest in pursuing gut models to evaluate the disposition and botanical-drug interaction potential of phytochemical constituents.

In silico modeling can also predict metabolites, essential for understanding NAM results as many *in vitro* systems do not have the same level of metabolic capability as humans. Analyses showed variability across commercially available prediction tools, with some compounds, like aristolochic acid, yielding consistent results, while others diverged, highlighting the need for further refinement. Physiologically based pharmacokinetic (PBPK) modeling using GastroPlus has also been employed to predict internal exposure, such as plasma and tissue concentrations, identifying high-exposure compounds to inform safety assessment.

In silico toxicity modeling was performed by most of the toxicity Working Groups. However, due to the number of models and the lack of ADME information in the tools, there were many flags for toxicity. By integrating ADME and in silico toxicity predictions, the team performed a pilot study to try to prioritize or deprioritize constituents based pairing in silico toxicity results with absorption information. Compounds with low absorption (<30%) are flagged as less relevant toxicologically as they are less likely to reach the target site, while those with high absorption (>30%) and predicted genotoxicity or hepatotoxicity are prioritized.

Future efforts can include looking at *in vitro* ADME tools, better predicting internal exposure, refining absorption and clearance evaluations, and expanding modeling capabilities.

## Dermal Toxicity

The Dermal Working Group is the most recently launched team of the BSC, starting in late 2023. It is focused on developing a screening strategy to evaluate potential dermal toxicity of botanicals, including skin irritation, skin sensitization and phototoxicity. By evaluating existing approaches for botanicals, the group aims to identify reliable tools for assessing dermal safety of these mixtures.

With the recent interest in botanicals used in cosmetics, the BSC has added additional botanicals that would be used in products not administered orally and thus needed to select new botanical case studies for this endpoint. The list included known sensitizing agents like poison ivy and essential oils known to be phototoxic (e.g., oil of bergamot).

Similar to genotoxicity, there are many mature *in vitro* assays that can be used for dermal toxicity, many of which include OECD Test Guidelines. For example, the group is exploring the use of the OECD 442 series (C-E) for skin sensitization and OECD 439 skin irritation assay.

Future efforts will focus on evaluating the proposed assays for testing using botanicals, with particular attention to skin irritation, sensitization, and UV-induced phototoxicity. As testing progresses, the group will refine its strategies. A strategy paper is in progress describing the selected assays and the list of botanicals relevant for dermal toxicity.

## Next steps and conclusions

Next steps for the BSC are to focus on evaluating the initial assays now that data are available, publishing results, and as resourcing allows, tackling the described next steps. Table 4 summarizes the progress and proposed next steps for each team. New areas, such as dermal toxicity, are being incorporated to broaden the applicability of the consortium’s work, while ongoing studies aim to refine assay batteries to improve sensitivity and specificity.

**Table 4.** Summary of the progress of BSC Working Groups, potential Next steps, and publications.

Working Group	Highlights of Progress		Potential Next Steps		Publications
Chemical Analysis	Sourced 16 botanicals Prepared extracts Supported the authentication of botanicals identified key constituents Published a strategy paper and a method manuscript on milk thistle		Publish additional method papers Expand botanical library Support data analysis for assays		Waidyanatha et al. 2024 Muchiri and van Breemen 2024 Sica et al. 2025
Literature Review	Developed a systematic review template		Utilize the template for additional botanical case studies		Patel et al. 2023
Data Analysis	Standardized data templates Developed Benchmark Dose Modeling pipeline		Continue BMD modeling and pairwise data analysis Analyze complex datasets Publish data pipelines Make data publicly available		
Hepatotoxicity	Selected botanical case studies Selected assays Generated data in the select assays using the botanical case studies Created a strategy manuscript Presented at scientific meetings	Published a workshop report Launched a comparison of botanical extracts and individual constituents for LDH release, MTT, and Cell Painting	Analyze assays data Benchmark assay to existing literature Publish results Collaborate with ADME, Data Analysis, and Chemical Analysis Working Groups to refine toolkit of assays	Expand assay battery Incorporate gut models for ADME studies.	Roe et al. 2025 Koturbash et al. 2024
Genotoxicity		Published an analysis of historical Ames Data		Add mammalian gene mutation assays Explore in silico models integrate ADME data Compare studies on individual constituents (e.g., aristolochic acids and aristolochia extract) to benchmark responses	Zeiger et al. 2024 Witt et al. 2025
DART		Selected additional botanicals relevant to DART		Expand botanical case studies Explore additional assays	Mahoney et al. 2025 (under review)
Cardiotoxicity				Test the botanicals in diverse cell lines Align with the Neurotoxicity Working Group to compare assays	Krzykwa et al. 2025
Neurotoxicity		Published a methods paper on the MEA assay with botanical Launched a comparison of botanical extracts and individual constituents for MEAs		Align with the Cardiotoxicity Working Group to compare assays.	Kanungo et al. 2024 van Kleef et al. 2024 Van Kleef et al. 2025 (under review)
ADME	Performed PBPK modeling Launched case studies on Absorption Clearance Metabolism		Refine tools for internal exposure predictions Publish case studies on in silico modeling Incorporate *in vitro* data with in silico predictions for ADME		Liu et al. 2024 Liu et al. 2025
Dermal Toxicity	Launched new group Selected botanicals relevant to toxicities Selected assays for skin irritation/sensitization and phototoxicity		Source and authenticate botanicals Evaluate assays using botanical case studies Publish strategy paper and results		Strategy paper in preparation

Future directions will emphasize cross-disciplinary collaboration to address gaps and improve the translational relevance of current assays. Efforts to integrate ADME data, chemical characterization, and toxicological modeling will enhance the interpretation of *in vitro* results and provide a better understanding of dose-response relationships. Benchmarking results against existing literature and advancing computational tools to account for complex interactions in botanical mixtures will be critical for ensuring that findings are robust and accurate. Additionally, targeted outreach and publications will help disseminate progress and engage with broader scientific and regulatory communities.

**Figure 2.**
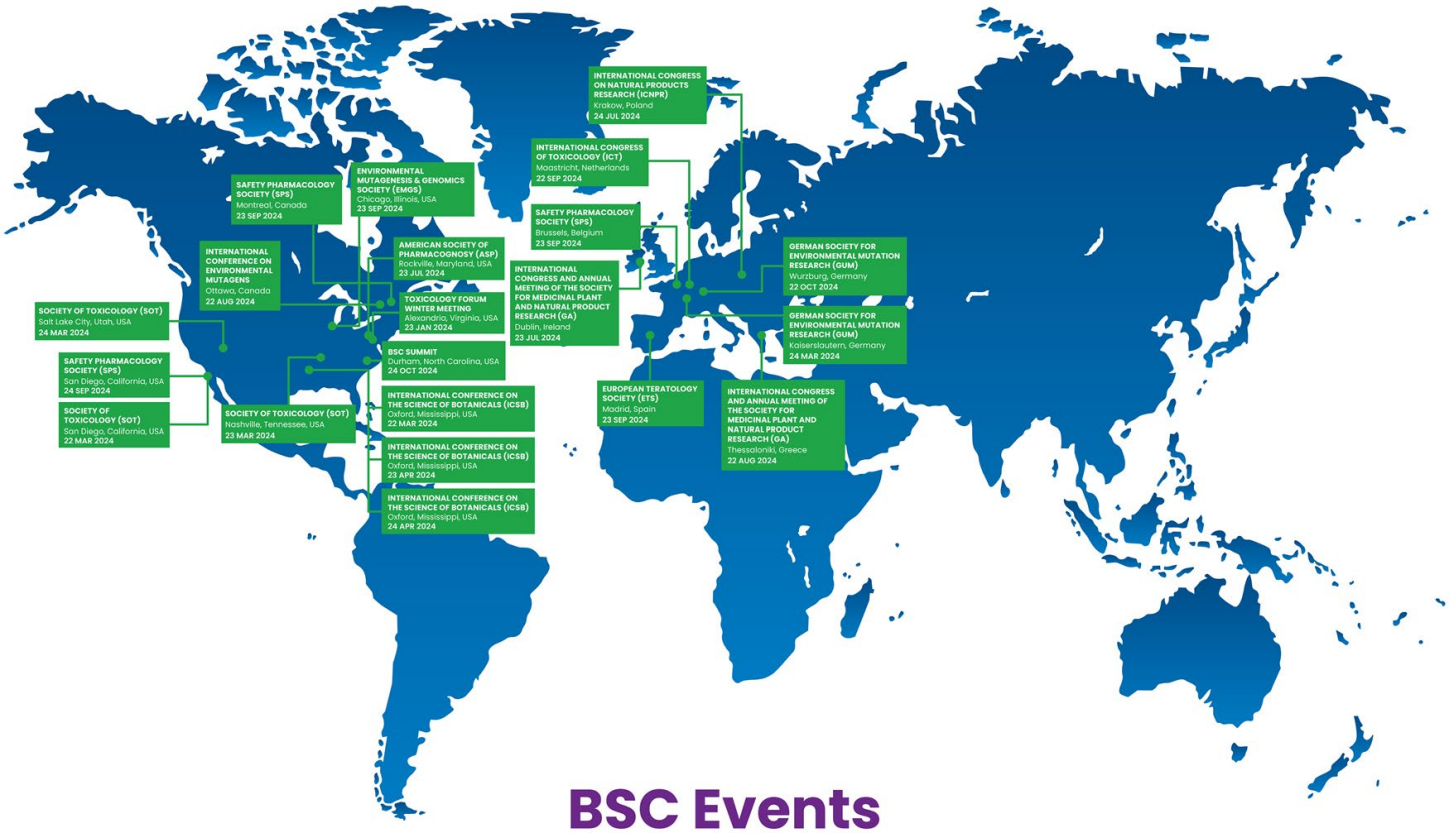
BSC events as of September 2025. A ‘living’ list can be found here.

The BSC has also participated in global outreach. In addition to sharing the results at many international conferences (Figure 2 e.g., Society of Toxicology, International Congress on Natural Products Research), the Steering Committee put together a training course entitled ‘Cultivating Safety: Toxicology 101 of Botanicals and Natural Products’ which is aimed at scientists in natural product research that want to learn and adopt the basics of toxicology. This course debuted at the 2024 ICNPR meeting in Krakow, Poland and will be presented at other scientific meetings in the future.

Additionally, we have partnered with groups around the world. After the COVID anti-viral treatments entered the market, we published a review paper on botanicals that may induce botanical-drug interactions (Smith et al. 2023). We also have an ongoing survey of botanicals taken by patients attending a diabetes and hypertension clinic in Blantyre, Malawi, led by investigators at the Kamuzu University of Health Sciences (Nyirenda et al. in press). An off shoot of this review is the quantification of aristolochia acid content of Aristolochia species found in the region as it was one of the species used by patients at the clinic. Both of these studies will be published in collaboration with the BSC network.

In conclusion, the BSC has made significant progress toward developing a cohesive strategy for botanical safety assessment that accounts for the complexity of botanical products. By leveraging advances in NAMs, chemical analysis, and data interpretation, the consortium is building a foundation for tools that researchers will be able to use for botanical safety. These efforts aim to support regulatory frameworks, improve consumer safety, and enable innovation in the development of safe botanical products.

## Data Availability

Data sharing is not applicable to this article as no new data were created or analyzed in this study
